# FinTech and financial instability. Is this time different?

**DOI:** 10.1080/01603477.2024.2315055

**Published:** 2024-04-01

**Authors:** Stefanos Ioannou, Dariusz Wójcik, Michael Urban

**Keywords:** FinTech, financial technology, financial instability, mixed methods, G20, G28, G50

## Abstract

We study the development of FinTech, defined as a set of innovations and an economic sector that apply recently developed digital technologies to financial services, with particular focus on payment and lending platforms, and digital asset management and online trading apps. We use mixed methods, including a theoretical exercise on the main balance sheet interactions involved in FinTech banking, and empirical insights from fieldwork in Latin America and the United States. Our analysis corroborates previous literature identifying several systemic risks in FinTech payment and lending platforms. These include the enhanced risk of a bank run, the increase in liquidity risk for incumbent banks, the fueling of precarious lending, and the potential compromise in the efficacy of monetary policy. Our discussion of online asset management and trading apps also highlights the risk of enhanced volatility in financial markets due to the increase in the participation of low-income and inexperienced investors. We observe that while the FinTech sector is still small in size, it already contains seeds of financial instability which should be all-too-familiar from recent history.

## 1. Introduction

Interest in the topic of FinTech has grown rapidly over recent years. A search in Google Trends reveals that on average in 2020 the word “FinTech” was searched in Google 40 times more often than in 2012 (worldwide). Some articles from the *Economist*, have described a FinTech revolution under way, rising against dinosaur banks that regulators refuse to let die (see respectively The Economist 2015, 2020). Likewise, Philippon (2018, 2) argues that FinTech can “revolutionize how existing firms create and deliver products and services, provide new gateways for enterpreneurship, [and] democratize access to financial services”. In recent years, FinTech has been embraced by national authorities as a vehicle for increasing competition in banking and for promoting financial inclusion. FinTech firms have also been targeted as a profitable outlet by institutional investors and venture capital (CCAF (Cambridge Centre for Alternative Finance)), 2021; Cojoianu et al. 2021).

Beneath the excitement lies a notable confusion in defining FinTech (Wójcik 2020a). Definitions vary, with some employing the term in the wider sense of “technology enabled financial solutions” (Arner, Barberis, and Buckley 2015) and others focusing more narrowly on “recently developed digital computing technologies applied to financial services” (Chen, Wu, and Yang 2019). Other scholars, such as Gomber, Koch, and Siering (2017), have established FinTech taxonomies, separating between functions (e.g. payments, lending, risk management), technologies (e.g. artificial intelligence, machine learning), and institutions (technology firms, financial services firms, etc.). Following the definition proposed in Wójcik (2020a, p.1), we define FinTech “as a set of innovations and an economic sector that focus on the application of recently developed digital technologies to financial services”.

Academic literature has engaged with FinTech in a variety of ways (for two comprehensive reviews see Wójcik 2020a, 2020b). Two themes that have gathered scholarly interest from critical social sciences are the relationship between FinTech firms and incumbent banks (e.g. Dermine 2016; Hendrikse, Bassens, and Meeteren 2018), and the discourse on financial inclusion (e.g. Aitken 2017; Bernards 2019; Gabor and Brooks 2017; Jain and Gabor 2020). Hendrikse, Bassens, and Meeteren (2018), for instance, show that contrary to narratives of disruption, incumbent banks have been increasingly active in developing FinTech incubators and accelerators across the world, so as to internalize benefits from new technologies and neutralize threats posed by FinTech firms.^1^ Bernards (2019) describes FinTech as a political phenomenon, inherently linked with the neoliberal approach to financial inclusion. Similarly, Aitken (2017) and Gabor and Brooks (2017) see FinTech as a new facet of financialization, associated with the administrative re-ordering of risky populations and the monetization of personal data.

Surprisingly, little research has been produced on the relationship between FinTech and financial instability, save for a discussion on cryptocurrencies (e.g. Zook and Blankenship 2018; Vogiazas and Alexiou 2019; an exception is Kregel 2016). This is a gap our paper aims to fill in. While FinTech as a sector is still too small to be visible from the macroeconomic point of view, there are several systemic risks that can be emerge as it grows in size.

We concentrate on two major branches of FinTech: payment and lending platforms, which we refer to as FinTech banking, and digital asset management and online trading apps. Payment platforms (also known as wallets) are the most basic product offered by FinTech firms. Lending platforms commonly separate into peer-to-peer (P2P) lending and balance sheet lending. Digital asset management and trading apps typically provide low-cost brokerage and asset management services, particularly in the form of robo-advising.

Our approach is based on mixed methods. For our analysis of FinTech banking (Section 4) we conduct a theoretical exercise in which we trace the main balance sheet interactions between FinTech, incumbent banks, the central bank and the household sector, and provide a contrast with the Post-Keynesian theory of endogenous money. We also utilize quantitative and qualitative data, throughout our paper. Our quantitative data is based on reports made available by the Cambridge Center for Alternative Finance (CCAF). Next to this, we consider a novel set of qualitative evidence from fieldwork research in US, Argentina, Brazil and Mexico, conducted between March 2019 and April 2020. Our fieldwork involved semi-structured interviews with high-profile professionals from the broader financial sector, including banks, FinTech firms, regulatory agencies, and advanced business services.

Due to the coverage of our fieldwork, our reflections are skewed toward the Americas, though also tied to the global level. If anything, the US FinTech industry is currently the largest in the world (CCAF (Cambridge Centre for Alternative Finance)), 2021), while San Francisco Bay is listed as the world’s lead FinTech hub (Findexable 2021).^2^ Similarly, several of the insights derived from our fieldwork in Latin America, as for instance the discussion related to financial inclusion, can be extrapolated in some part and considered in the context of other emerging and developing economies. Consideration of BigTech firms lies outside the scope of our paper, given that the size of these companies makes them incomparable with other FinTech firms (for discussion on BigTech see FSB (Financial Stability Board) 2019b and Cornelli et al. 2020).

We proceed as follows: in the next section we provide a brief overview of the macroeconomic environment within which FinTech develops. Next, we discuss our methodology. We then delve into FinTech banking. Following, we discuss digital asset management and trading apps. In the last section we summarize and conclude.

## 2. The broader macroeconomic background

As defined here, FinTech is by and large a product of the late 2000s and the 2010s. Its genesis falls closely to the invention of the smartphone and the US and European crises of 2007–2010. As a result of these crises, this was a time of massive injections of capital to support failing too-big-to-fail (TBTF) banks, flooding of banks’ balance sheets with liquidity via Quantitative Easing, steep cuts in interest rates, and an attempt to re-introduce certain pieces of financial regulation, such as prohibition of proprietary trading and establishment of resolution schemes for failing banks (see amongst others Taibbi 2012; Pollin 2012; Seccareccia 2017; and Ioannou, Wójcik, and Dymski 2019). It was also an era of significant rises in unemployment. In the US, the rate of unemployment more than doubled as a result of the crisis, hiking from 4.4% in 2006 to 10% in 2009 (source: Fred).

In Latin America, FinTech emerged in an environment traditionally characterized by high banking centralization, a relatively high degree of financial regulation, and significant political and economic instability (Ioannou and Wójcik 2021). Out of countries with notable FinTech presence, Argentina (6th in development of FinTech in Latin America according to CCAF (Cambridge Centre for Alternative Finance), 2021) is the most representative example of economic and political turbulence during the 2010s, having experienced significant currency instability, steep inflation, and a sovereign debt crisis in 2014 (Laeven and Valencia 2018; Valdecantos 2020). Latin American countries have also been characterized by low levels of financial inclusion. According to World Bank (2018), the share of adult population with a bank account was 55% in 2017 for the whole region (including the Caribbean). Chile and Brazil were at the higher end, with 74 and 70% respectively, while at the other end, Mexico and Peru registered 37 and 43%.

Macroeconomic shortcomings have often led to advocacy of FinTech as a remedy by regulators who have embraced FinTech as means to improve transparency, efficiency, competition and inclusion in banking (FSB (Financial Stability Board)), 2017, 2019a; Carney 2017; World Bank and CCAF (Cambridge Centre for Alternative Finance), 2019).^3^ In the US, some FinTech entrepreneurs have presented their companies as alternatives to the banks responsible for the 2007/08 crisis, even claiming inspiration from the demands of the Occupy Wall Street Movement (CNN Business 2017). Others in Latin America have claimed to be after those “doomed to use cash” (interview INT_4 of ours). Such voices, however, are mostly expectations than facts. Rather than detecting a “battle” between FinTechs and incumbent banks, CCAF (Cambridge Centre for Alternative Finance) (2021) finds that trust to traditional banking institutions is positively correlated to the growth of FinTech. According to the same report, FinTechs in Latin America have mostly sought the custom of those already banked.

## 3. Methodology

We employ mixed methods. For the analysis of the various models of FinTech banking, we conduct a theoretical exercise to analyze the basic balance sheet interactions between FinTech, incumbent banks, the central bank and the household sector, and contrast these interactions with the Post-Keynesian theory of endogenous money.

On the empirical front we utilize quantitative data made available by CCAF. To the best of our knowledge, CCAF is one of the richest sources of data on FinTech and provides free online access. Its data is based on standardized surveys of hundreds of FinTech representatives from around the world.

Next to quantitative evidence we consider a novel set of primary data from fieldwork research in the United States, Argentina, Brazil and Mexico, conducted between March 2019 and April 2020. Our fieldwork involved interviews with high-profile professionals from finance (including FinTech) and advanced business services, as well as high-level officials from regulation in each of these countries. In total we conducted twenty-seven interviews. Nine were with FinTech professionals, and the rest with professionals with whom we also discussed FinTech (Appendix Table A1). With the exception of our interviews in Argentina and Mexico, which due to the covid-19 pandemic were conducted online, all others were conducted face-to-face. In all cases, interviews lasted about an hour, and were recorded and transcribed.

All interviews were semi-structured (Clark 1998; Longhurst 2010). Prior to each, we shared a list of broad topics with our interviewees, which were centered around the evolution of the region’s leading financial centers, such as New York and São Paulo, and other issues, related to financial stability, the nexus between finance and politics, as well as the development of FinTech. Given this orientation, we also allowed our interview partners to drive the conversation, whenever we deemed this was beneficial to our research. This strategy was particularly valuable as it allowed each interview partner to spend more time on issues they were most familiar with and considered most important. For arranging interviews, we used our own networks, as well as corporate websites and LinkedIn. Our target in terms of seniority of interviewees was the executive level, whenever possible. The names of interviewees, details of their organizations, and precise locations of interviews have been anonymised.

 In terms of correspondence with the rest of the paper, our theoretical exercise provides the focal point of Section 4.2 while our empirical material informs the whole of the paper. In consideration of space constraints, we only use interview extracts selectively.

## 4. FinTech payment and lending platforms

### 4.1. Basic characteristics

The most basic type of FinTech firms are payment platforms. Most commonly, these platforms offer deposit accounts, typically linked with money transfer and payment services. These are usually advertised as more user friendly, cheaper and involving less onerous bureaucratic procedures, compared to accounts offered by incumbent banks. Examples of FinTech companies offering such products are Chime in the US, Nubank in Brazil, and Ualá in Argentina.

FinTech lending predominantly separates into peer-to-peer (also known as marketplace) lending and balance sheet lending. In its basic form, the peer-to-peer (P2P) model aims at the channeling of funds and information between savers and borrowers, without any withholding of funds by the platform. This is in contrast to balance sheet financing where the intermediating FinTech is allowed to provide loans to its customers.

**Figure 1.**
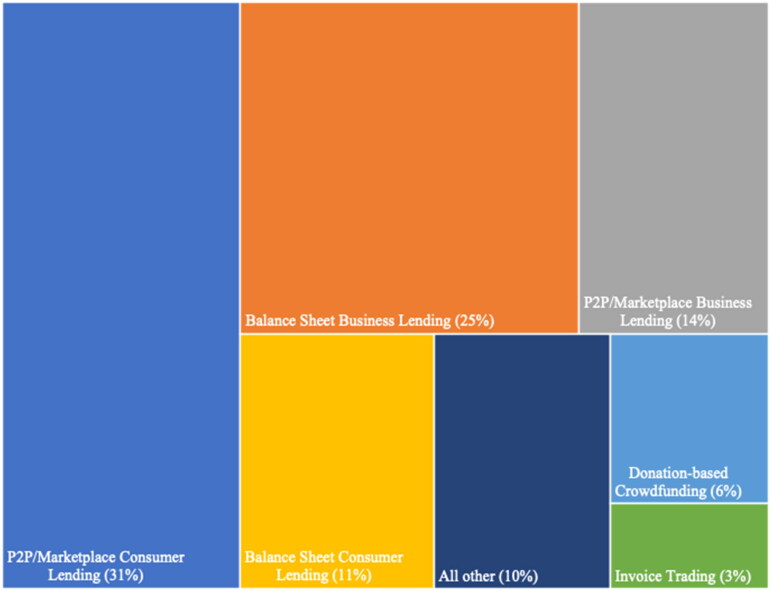
Global shares of FinTech financing models out of total global FinTech financing for 2020. *Source*: CCAF (Cambridge Centre for Alternative Finance) (2021).

According to CCAF (Cambridge Centre for Alternative Finance) (2021), and as illustrated in Figure 1, 45% of global FinTech lending takes the form of P2P lending (50 billion USD), followed by balance sheet lending with 36% (40 billion USD). Other categories of FinTech financing include donation-based crowdfunding (6%) and invoice trading (3%).

The actual FinTech firms providing lending are often firms originally established as payment platforms, which have organically grown in size. The transition of FinTech firms from simpler to more complex business models, often involving a higher element of risk, was indeed a common observation that emerged in our fieldwork in Latin America and the US (INT_1; INT_2; INT_3; INT_4; INT_10; INT_16; INT_24). A representative of a Mexican FinTech, for example, described how they started as a crowdfunding platform, subsequently moved into deposit business, and were, at the time of the interview (March 2020), planning to pilot loan offering (INT_16). The following quote, from a senior director of a large Argentinian FinTech, is a notable manifestation of this trend (INT_1):

The way I put it is, you open a door and there’s two doors, we had that one door and then you open it and it goes two doors and there is another two doors. You come into opportunities, we started with […] payments online, payments with POS, payments with QR, and then we started offering credit and then we started offering money market investments. […] Something similar is happening in Brazil […] and something similar is happening in Mexico.

It is also important to note that several of the differences in payment and lending FinTech models result from differences in regulation rather than technology. A complicating factor is the fact that regulatory frameworks for FinTech banking vary significantly across countries (Ehrentraud, Garcia Ocampo, and Quevedo Vega 2020). In some cases, FinTech firms can be licensed in a way that allows them to keep deposits on their own balance sheet. In certain states in the US, for example, FinTech firms can be registered as industrial loan companies (ILCs) - in other words as institutions, owned by a non-financial parent company, that can accept deposits covered by the Federal Deposit Insurance Corporation (FDIC), and provide loans. Similarly, FinTech firms in Mexico can choose to register as Sofipos (acronym for “Sociedades Financieras Populares”), a rough equivalent of a regional bank with a specific purpose, such as financial inclusion (CNBV (Comisión Nacional Bancaria y de Valores) 2020).

In practice, however, FinTech firms to register as such are few in number due to the enhanced regulatory requirements (e.g. capital requirements) and supervision that come as a package. This observation was also confirmed in our interviews (INT_16; INT_17; INT_20; INT_27). In the absence of such licensing, FinTechs are typically required to either deposit their funds in accounts of incumbent banks or at the central bank.

### 4.2. From endogenous money to FinTech banking

According to the basic tenets of Post-Keynesian economics, money supply is endogenous in the sense of being created by commercial banks, rather than set exogenously by the central bank (Fontana 2009; Tymoigne 2018; Lavoie 2019). Contrary to loanable funds theory, according to which banks use preexisting savings to provide credit, Post-Keynesian theory suggests that when granting loans, banks generate money *ex nihilo*. Credit money takes the form of bank deposits and is backed up by central bank reserves, the latter made available upon request by the banks *after* new money has been created. In turn, the interest rate on reserves is the basic tool by which a central bank exercises monetary policy in normal times.

The first part of Table 1 illustrates by means of a simple example how money is created according to Post-Keynesian theory. Following the application and approval of a loan of 100 USD, there are four entries generated simultaneously in the balance sheets of the bank and the borrower (call her Elena). The loan registers as an asset for the bank and a liability for Elena. Likewise, the deposited funds made available to Elena mirror reflect as a liability for the bank. Only after the generation of the loan, the bank is required to increase its holding of central bank reserves, to comply with bank regulation (if need be).

**Table 1.** Balance sheet interactions in FinTech banking.

			Central Bank		Bank A		FinTech platform		Elena		Wholesale lenders (part 5); John (part 6)		Potential systemic risks related to FinTech
Part	Scenario	Steps	Assets	Liabilities	Assets	Liabilities	Assets	Liabilities	Assets	Liabilities	Assets	Liabilities	
1. Endogenous money.	S0	1. Creation of money.			Elena’s loan (100)	Elena’s account (100)			Account in bank A (100)	Loan (100)			
		2. Acquisition of reserves (here by selling Treasury bills to the central bank).	Treasury bills (10)	Bank A's account (10)	Treasury bills (−10)								
					Reserves (10)								
2. FinTech platforms licensed as payment institutions, but required to store funds in an incumbent bank (e.g. Uala in Argentina); or in strategic collaboration with incumbent banks (BaaS) (e.g. Chime in the US).	S1	1. Opening of bank account in FinTech platform.				Elena’s account (-50)			Account in bank A (-50)				Risk of bank run due to lack of deposit guarantee; risk of reputational contagion.
						FinTech platform’s account (50)	Account in bank A (50)	Elena’s account (50)	Account in FinTech platform (50)				
3. FinTech platforms licensed as payment institutions with account with the central bank, e.g. Nubank in Brazil.	S1	1. Opening of bank account in FinTech platform.		Bank A's account (-50)	Reserves (−50)	Elena’s account (−50)	Reserves (50)	Elena’s account (50)	Account in bank A (−50)				Risk of bank run due to reputational contagion; risk of adverse interaction with sovereign credit profile; liquidity risk for incumbent banks.
				FinTech platform’s account (50)					Account in FinTech platform (50)				
		2. Store of deposits in Treasury bills (the case of Brazil).	Treasury bills (−50)	FinTech platform’s account (−50)			Reserves (−50)						
							Treasury Bills (50)						
4. FinTech platforms licensed as banks, e.g. Square in the US (licensed as an ILC); Brubank in Argentina.	S2	1. Creation of money.					Elena’s loan (100)	Elena’s account (100)	Account in FinTech platform (100)	Loan (100)			Risk of lending fragmentation by type of lender if FinTech platforms target marginalized borrowers; risk of loosening of lending standards by incumbent banks to cope with competition; risk of rise in financial fragility for households; risk of compromise in the efficacy of monetary policy (part 5).
		2. Acquisition of reserves (here by selling Treasury bills to ^the^ central bank).	Treasury bills (10)	FinTech platform’s account (10)			Treasury bills (−10)						
							Reserves (10)						
5. FinTech platforms licensed as non-bank lenders, e.g. Rocket Mortgage in the US.	S2	1. Raise of funds via wholesale funding.				Wholesale lenders’ accounts (−1000)					Account in bank A (−1000)		
						FinTech platform’s account (1000)	Account in bank A (1000)	Debt to wholesale lenders (1000)			Loan to FinTech (1000)		
		2. Loan of 100 USD to Elena based on own resources.				FinTech platform’s account (−100)	Account in bank A (−100)						
						Elena’s account (100)	Loan to Elena (100)		Account in bank A (100)	Loan (100)			
6. FinTech peer-to-peer (marketplace) lending platforms, e.g. Lending Club in the US until 2020; Prestadero in Mexico.	S3	1. Loan of 100 USD to Elena via peer-to- peer platform.				John’s account (−100)					Account in bank A (−100)		Risk of loosening of lending standards a) by incumbent banks to cope with competition and b) by P2P platforms due to absence of diligent monitoring; credit risk passed to investors.
						Elena’s account (100)			Account in bank A (100)	Loan (100)	Elena’s loan (100)		

*Source*: authors’ elaboration. *Notes*: bank A set as an example of an incumbent bank; Elena set as an example of a FinTech customer and John as an example of a peer-to-peer lender; sums of USD in brackets.

List of scenarios: S0: endogenous money (default scenario). Bank A makes a loan of USD 100 to Elena; S1: Elena opens an account in a FinTech platform and transfers some of her deposits (USD 50) from her account with bank A; S2: Elena takes up a loan of USD 100 from a FinTech; S3: John finances Elena’s startup via a P2P lending platform.

While simple, the above example is a useful point of reference for studying the differences between traditional banking and the various models of FinTech banking. To this end, the rest of Table 1 elaborates analytically the various FinTech models from a balance sheet perspective.

We start with the consideration of FinTech payment platforms that are required to store deposits in an incumbent bank. This could either be a regulatory obligation, as in Argentina, or due to strategic collaboration of the FinTech with an incumbent bank. An example of the latter scenario is the Banking as a Service (BaaS) business model, wherein a FinTech uses a bank’s license and digital infrastructure to provide banking services and products, either by co-branding them or by offering them under its own brand. Part 2 of the table provides a simple illustration of what happens when a customer (Elena) decides to open a new bank account with such a FinTech and deposit 50 USD. There are two new entries of 50 USD at the balance sheet of the FinTech platform, one on the liability side, corresponding to the balance in Elena’s bank account, and one on the asset side, corresponding to the balance of FinTech’s account in the incumbent bank A.^4^ The incumbent bank A debits 50 USD to Elena’s account and credits the same amount to the account of the FinTech. At the aggregate level, bank A’s balance sheet remains unchanged. Likewise, the size of Elena’s balance sheet also remains unchanged as the only change recorded is a reshuffling of funds in the asset side, away from Elena’s account in bank with A and toward her newly opened account in the FinTech platform.

Despite the simplicity of the above operation, a potential systemic risk opens up due to the transferring of deposits away from banking institutions that would be typically covered by a deposit guarantee scheme. Whether the deposits in the receiving FinTech also enjoy a similar protection is a case-by-case matter. In Argentina, for example, payment platforms are (at the time of writing) required to explicitly disclose in their websites that their deposits do not constitute deposits in a financial institution, nor do they have any protection as with bank deposits (see Ualá 2023 for an example). In the US, FinTechs based on the BaaS model, such as Chime, have to declare that their deposits are held at FDIC-insured banks. Although an indirect form of protection, there is still ambiguity as to whether such deposits are indeed protected, considering the de jure ceiling in deposit guarantee protection (250,000 USD in the US).

Protection status aside, FinTech payment platforms have a further disadvantage compared to banks, the fact that their reputation is still untested. That means that FinTech platforms can be more susceptible to a bank run due to reputational contagion, if financial distress of one FinTech gets to be interpreted as indicating distress across the FinTech sector as a whole (FSB (Financial Stability Board) 2017).

A second category of FinTech payment platforms are those allowed to have an account with the central bank. Brazil is an interesting case to consider in this context, due to its legislation to recognize FinTech payment platforms as a distinct type of non-bank payment institutions. According to this legislation, FinTech payment platforms are allowed to open a specific type of bank accounts, called ‘payment accounts’, and accept deposits (Banco Central do Brasil, 2019; Nubank 2023). While funds deposited in payment accounts do not enjoy the usual deposit guarantee of bank accounts, FinTechs are required to store them in Treasury bills, so that in effect, their risk is equivalent to the risk of default of the federal government of the country.

Part 3 of Table 1 illustrates the mechanics involved when Elena decides to open an account with such a FinTech and transfer 50 USD from her account with bank A. While the scenario is the same as before, there is now a major difference. By attracting Elena’s deposits, the FinTech’s balance sheet now increases at the expense of the balance sheet of bank A.

Naturally, this scenario is consistent with the expectation of enhanced competition in banking, a prospect long awaited by bank regulators, particularly in regions with a high degree of bank centralization as in Latin America (World Bank and CCAF (Cambridge Centre for Alternative Finance), 2019). On the downside, a significant move of deposits from incumbent banks to FinTech platforms runs the risk of augmenting liquidity risk and funding volatility for banks (Carney 2017).

In the context of developing countries like Brazil, another risk involved in the policy of obliging FinTech platforms to store their deposits in Treasury bills is the enhanced possibility of a “doom-loop”, wherein a deterioration of the credit profile of the central government leads to a bank run on Fintech platforms, which in turn feeds into banking instability and further deteriorates the credit profile of the government (see Gibson, Hall, and Tavlas 2017 for a similar argument on the interaction between bank credit ratings and sovereign credit ratings).

Parts 4–6 of Table 1 describe three alternative varieties of FinTech lending, covering FinTech balance sheet lending (parts 4 and 5) and P2P lending (part 6). In the case of balance sheet lending the distinction between parts 4 and 5 is on the basis of whether FinTech firms hold a banking license or not. We first outline the mechanics corresponding to each part and following, we discuss the systemic risks that can emerge as FinTech lending grows.

Part 4 elaborates the scenario in which lending FinTechs are licensed as banks. Here, the analytical steps involved in loan creation are similar to the scenario of endogenous money (part 1 of the table). Following the approval of the loan, there are four entries generated simultaneously in the balance sheets of the FinTech and the borrower. As with endogenous money, the FinTech does not lend anything it possesses beforehand, hence there is no decline in any of its assets when granting the loan. Following that, the FinTech needs to access central bank reserves to comply with regulation, either by purchasing or borrowing them (as with the first part we assume the FinTech purchases reserves by selling Treasury bills to the central bank). Whilst there might be regulatory differences as to how much reserves and capital a licensed FinTech needs to hold, the basic mechanics are the same as with money creation by incumbent banks.

Part 5 of Table 1 illustrates what happens when a FinTech licensed as a non-bank lender (e.g. Rocket Mortgage in the US) grants a loan. A major difference with the previous part is that non-bank lenders can only provide loans based on their own resources, hence, the first step is to raise funds. This could take a variety of forms, such as wholesale funding or attraction of venture capital. Here, we pick the first scenario as an illustration. Assuming an originally idle state for wholesale lenders’ funds, the first step involves the transferring of funds (say 1,000 USD) within bank A, from the bank account of wholesale lenders to that of the FinTech platform. On the side of the FinTech, there are two entries of 1,000 USD that simultaneously appear on its balance sheet, one in its asset side, to record the availability of new funds, and one in its liability side, to record its debt to wholesale lenders. Further, wholesale lenders’ asset side records two changes, first, a decline in their available deposits by 1,000 USD, and second, a new asset of equal nominal value, corresponding to their loan to the FinTech. Effectively, the main implication for them is the trading of a more liquid asset (deposit money) for a less liquid asset (loan to the FinTech), in the expectation of a higher return.

Once funds are raised, the FinTech platform can go ahead and grant the loan to Elena, who has just applied for a mortgage. On the side of Elena’s balance sheet, the entries are the same as before, with the newly acquired funds in the asset side and her loan commitment in the liability side. For the FinTech platform, the granting of the loan involves the transferring of funds from its own account in bank A to that of Elena (being a non-bank lender, we assume the FinTech cannot hold deposits in its own balance sheet). While the total asset size of the FinTech remains unchanged, the firm has traded a liquid asset (100 USD of deposit money) for a less liquid asset (Elena’s loan of 100 USD), with the expectation of a higher profit, similar to the case of wholesale lenders.^5^

Part 6 elaborates the most basic variety of P2P lending. Here, the FinTech platform performs a pure intermediation role between borrowers and savers, typically by inviting the first to make a loan application (accompanied by a business project proposal to justify the use of funds if the P2P platform specializes in business lending) and, following, by presenting a lending “menu” to savers.^6^ Save for a commission fee (omitted in Table 1), the P2P FinTech does not record any entries on its own balance sheet but only facilitates the relocation of funds from the savers’ to borrowers’ accounts, while enabling the generation of a new asset (liability) for the saver (borrower). The lower part of Table 1 provides a simplified illustration on the assumption of a single saver (John) and borrower (Elena), and considering again a loan of 100 USD (it is also possible to interpret John and Elena’s balance sheets as representing the aggregate balance sheets of P2P lenders and borrowers).

There are three types of risks that can emerge out of the above varieties of FinTech lending. First, FinTech lending can fuel precarious lending, either indirectly or directly. Indirectly, the intensification of competition with incumbent banks can push the latter to cut their lending standards so as to preserve their profitability and satisfy their shareholders (FSB (Financial Stability Board) 2017, 2019a). For example, banks might become more tolerant to higher debt-to-income ratios for new borrowers, thereby enabling the expansion of lending to previously unqualified low- income borrowers. Cornaggia, Wolfe, and Yoo (2018) provide some corroborative evidence for the US, showing that the expansion of P2P lending led to a significant loss of loan volume for small commercial banks, particularly in rural areas.

Directly, FinTech lending platforms, whether balance sheet or P2P, can fuel precarious lending either by lending funds to unbanked households with no credit history, or by enabling low-income households to refinance their debts. For the US, Buchak et al. (2018) and Fuster et al. (2019) confirm that FinTech mortgage lenders have mostly been focused on debt-refinancing. Tang (2019) shows that US FinTech firms have mostly attracted borrowers already included in the banking system, but from the lowest creditworthiness levels. Likewise, Jagtiani and Lemiuex (2018, 2019) argue that FinTech firms have attracted borrowers classified as subprime by traditional lending criteria. They also show that FinTech firms have penetrated areas underserved by traditional banks and areas with poor economic performance.

Our evidence from Latin America suggests a similar picture. In Argentina, an interviewee from a consulting firm confirmed that FinTech firms are targeting low income households and millennials (INT_5). In Brazil, several interviewees also confirmed that FinTech firms’ expansion in the country has been toward both low income households and millennials (INT_11, INT_9, INT_10). In Mexico, an interviewee from a bank lobbying institute (INT_17) mentioned that FinTech firms are predominantly after people with no credit history. As (s)he put it in our conversation:

If I were those credit FinTech companies, I think I did not care a lot of how to calculate the risk because with that kind of return of money, with that interest, if one of four clients don’t pay me, I still have winnings, earnings. […] The average [interest rate on credit cards] is 10 times bigger than the bank. […] But they lend you the money in 15 minutes. So, a lot of people are using these FinTech firms when they have a money problem, I don’t know, maybe medical problem, a funeral or […] because they were fined and they need the card to have more money…

At the global scale, Claessens et al. (2018) offer some mixed evidence, confirming that FinTech lending to households has been mostly used for debt-refinancing, while also noting that the high default rates relative to banks recorded in several countries, including the US, indicate that part of FinTech lending might have also been targeting marginal borrowers.

A further risk is the expansion of securitization. The securitization of FinTech-generated mortgages has already been recorded as a common practice in the US (Buchak et al. 2018; Fuster et al. 2019).^7^ Typically, FinTech mortgages are securitized based on guarantees from government-sponsored enterprises like Fannie Mae and the ‘originate and distribute’ model—a model well known for the weak risk-monitoring incentives it produces for the loan originator (Kregel 2016; Navaretti, Calzolari, and Pozzolo 2017). Following, FinTech platforms offload their loans onto a “FinTech” vehicle (FinTech in the sense of being heavily reliant on artificial intelligence and big data analytics), which, in turn repackages them and sells them to hedge funds, private equity funds, banks and others (Hughes 2017; Gara 2019; Omarova 2019).

Third, an expansion of FinTech lending can compromise the efficacy of monetary policy (FSB (Financial Stability Board), 2019a). Most notably, the inability of non-bank balance sheet lenders and P2P lenders to transact directly with central banks means that these types of lenders are deprived of access to liquidity facilities of central banks and are not liable to any reserve requirements. At one level, this implies that central banks have less control over the operations of such platforms in normal times. It also means that central banks are unable to intervene as lenders of last resort in times of turbulence, an aspect which further amplifies the risk of reputational contagion. Compared to non-bank balance sheet lending, these risks are even more acute in P2P lending due to the decentralization of credit supply, and the challenge involved in tracing individual credit risk exposures of P2P lenders. Decentralization also makes it more challenging to design effective resolution mechanisms for P2P platforms (Financial Conduct Authority (FCA) 2018).

## 5. Digital asset management and trading apps

Robinhood, a trading app that offers zero-commission trades and a simple, video-game-style interface, had 3 million new accounts opened in the first quarter. Half of its new customers are first-time investors. Many online communities are filled with […] stories of fabulous fortunes gained, hot tips, trading systems and theories and so on. […] day traders often don’t understand the amount of risk they’re taking. […] One young novice investor tragically committed suicide after seeing his account generate large losses. (Smith 2020)

The second major category of FinTech to consider in this paper are digital asset management and trading platforms. Typically, these platforms enable the investment in financial markets for a wider population, particularly middle class, millennials, and low-income households. This can happen in two ways, either by a FinTech providing low-cost brokerage or by offering asset management services, particularly in the form of robo-advising. Robinhood, a US trading platform with current capitalization of about 8.5 billion USD as of May 2023 is a prime example of a FinTech that enables its users to trade in stocks, gold, cryptocurrencies and options. In robo-advising, FinTech apps tailor an investment portfolio for the users, based on their preferences, such as risk-sensitivity and investment horizon, while an algorithm executes trades, based on these preferences.

Viewed against the established practice of megabanks setting seven-digit money floors for providing asset management services, FinTech has enabled what at first sight appears as the “democratization” of the industry. In actual fact, however, rather than enabling the access to financial markets in equal terms, FinTech is more likely to widen the gap between high- and low-income investors, in terms of their expected returns from the market, and the volatility of those returns.

Consider, first, the inherent risk of robo-advising to augment herd behavior and procyclicality (Magnuson 2018; Omarova 2019; Carney 2017). As pointed out by Omarova (2019, 789), “super-fast trades being executed via robo-advisors’ algorithms […] are likely to form potentially highly correlated tidal waves of money moving in and out of the same asset classes”. This is even more so in consideration of the high degree of centralization observed in the robo-advising industry. Haberly et al. (2019) observe that the rise of FinTech has produced a winner-takes-all paradox of ‘centralization through democratization’, where major financial firms have come to benefit through incremental innovation and the take-over of smaller competitors.

Consider, secondly, that human interaction and professional investment advice still matter for high-income investors, corporate customers, banks and funds, despite advancements in technology. Interviews INT_19 and INT_21 from our fieldwork provide some indicative corroborative evidence of this trend. As expressed by INT_19 from an international investment bank:

We still have lots of clients […] that really want to trade and interact with a human on the other side. Because it’s of their best interest to negotiate the price, to negotiate the conditions […] to make the trade more tailor made, you know? So, for the retail banking, of course the people will feel comfortable just clicking and trading FX from their own bank portal, but for corporate and foreign banks, for big pension funds or asset managers, they […] will always want to have a human in order to make things like, for example, the FX [foreign exchange] trade, they are not going to the platform and do it. If they are going to trade volume, they want to speak to someone and tell him, ‘I will trade $200 million in one FX trade, what price are you going to offer me?’ They don’t want a machine or they don’t want a flat app platform dictating which price they are going to close the trade at.

To be sure, the enduring reliance on human interaction is not to say that high-income households do not benefit from advancements in financial technology. They do so, but in rather a different way, via the exploitation of new technologies, such as artificial intelligence and machine learning, by the professional asset managers who serve them. An illuminating testimony how artificial intelligence is used in this context comes out of an interview of ours with a Mexican asset management firm (INT_18):

…instead of forecasting inflation […] we decided to measure prices so we built robots and what these robots do, for example, they open an account in your local Walmart, we download every single product […] then we log out, log back in again but instead of saying to Walmart that we are in City 1, we’re now in City 2, and we repeat this process across all supermarkets and all kinds of providers […] every basis point that we beat the market, I don’t know, like a normal trading strategy can give you like $1m of earnings or something like that […] we’re now in the next stage because we became so good at this that we’re not only capable of forecasting inflation better than anyone, but we can also do some kind of reverse engineering. So, we know or we think we know the way Citigroup is thinking and Goldman Sachs is thinking and Morgan Stanley’s thinking, so we can forecast their forecasts before they do. So that’s put us in a very good position for trading and for our mutual funds. Those are the kind of things we’re doing for everything, for stocks for bonds, for currencies….

A financial forecaster in the US also gave us a similar example (INT_25):

we use machine learning to identify key words and phrases that are predictive of events, so the first one we started with was, “to file for bankruptcy”, so we have a credit risk model that looks at unstructured text, so during a quarterly conference call, a sell side analyst asks the CFO, “Is there any risk of you violating your debt covenants?”, that would be one of those key words and phrases that are highly correlated with default because that’s not a question that’s typically asked on an earnings call, so it matters less what the answer is- just the fact that there’s a company expert out there who’s worried enough to ask, produces a red flag.

These observations indicate that FinTech can mean different things to different groups of people. On the one side, FinTech as a new set of apps enabling online trading predominantly address low-income and inexperienced investors. On the other side, FinTech as a new set of advanced technologies to aid market analysis and forecasting, stands to empower further high-income investors, thereby cementing their advantageous positioning vis-à-vis petit investors. Moreover, the entrance of a new crowd of inexperienced investors in financial markets stands to amplify volatility in financial markets and expand the reach of the repercussions stemming out of financial bubbles and crashes.

## 6. Conclusion

Our paper aims at exploring the links between FinTech and financial instability. While the FinTech sector is still small in size, it has grown significantly over recent years in many parts of the world, even in face of the COVID-19 pandemic (Wójcik and Ioannou 2020). It is thus likely to grow further into the near future. Our discussion focuses on FinTech payment and lending platforms, and digital asset management and trading apps. For our analysis, we use mixed methods, combining a theoretical analysis of the main balance sheet interactions between FinTech and the rest of the economy with quantitative and qualitative data, the latter based on fieldwork research in Latin America and the US.

Our balance sheet analysis illustrates the systemic risks involved in FinTech banking. First, the lack of an explicit deposit guarantee and the still untested reputation of the FinTech sector amplify the risk of a bank run on FinTech deposit platforms. Secondly, the extraction of deposits from incumbent banks amplify liquidity risk and funding volatility for banks. Third, FinTech lending can fuel precarious lending, indirectly, by pushing incumbent banks to compromise further their lending standards, and directly, by accommodating unbanked and low-income households. FinTech lending also stands to aid further the expansion of securitization, a financial practice closely associated with the US crisis of 2008 (Dymski 2010). Forth, it risks compromising the efficacy of monetary policy due to the lack of access of several types of FinTech payment and lending platforms to the liquidity facilities of central banks and the decentralization of credit supply in P2P lending (Financial Conduct Authority (FCA) 2018; FSB (Financial Stability Board), 2019a).

Our discussion of digital asset management and trading platforms confirms the uneven accessing to financial technology by low- and high- income households. On the one side, the enabling of easier access to financial markets for low-income households and the algorithmic management of their savings by robo-advising platforms amplifies their exposure to herd behavior and pro-cyclicality risks. This contrasts with high-income households who stand to gain the most out of the use of advanced technologies, such as artificial intelligence and machine learning, by the professional asset managers who serve them. Taken together, these developments stand to amplify volatility in financial markets and expand the reach of financial bubbles and crashes.

To be sure, our aim is not to deny the merits of FinTech and its potential to aid financial development, stability and supervision (Kregel and Savona 2020). Regulators’ envisioning of the use of RegTech (regulation technology) for real-time monitoring of financial information and capital flows is indicative of ways in which technology could be used to such end (Haldane 2014; Arner, Barberis, and Buckley 2017). Likewise, the easier collection of high-quality information, such as data on transactions, could help banks make more informed lending choices, and thus reduce rates of default. Rather, our aim is to comment on the ways FinTech has been developing within a specific socio-economic environment, still characterized by inadequate financial regulation, and financial practices such as securitization continuing to resemble the pre-crisis era (Ioannou, Wójcik, and Dymski 2019).

There are several limitations in our work. First, our paper does not comment on the impacts of the COVID-19 pandemic on the FinTech sector. While early evidence suggests the continuing growth of the sector, it is still too early to know how future events will play out. Second, our omission of China, a country with the largest FinTech sector until recently, limits the possible degree of generalization of our observations. Similarly, our focus on Latin America only allows for a limited extrapolation to other parts of the developing world, such as Africa. The consideration of FinTech in the context of a stock flow consistent (SFC) model would also be a natural extension of the balance sheet analysis provided in this paper. Each of these limitations is fertile ground for future research. Overall, we hope our paper will encourage further engagement of heterodox economics with the controversial topic of FinTech.

## Footnotes

1 Interviews of ours were also affirmative of the increasing importance in the collaboration between incumbent banks and FinTech firms (INT_14; INT_15; INT_26).

2 China was until 2019 the largest country in terms of volume of FinTech activity, but with a steady decline since 2017, due reported cases of fraud and regulatory toughening (CCAF (Cambridge Centre for Alternative Finance), 2021).

3 In our interviews with officials from regulatory agencies, we also recorded similar remarks (INT_12; INT_13).

4 Hereafter we consider just one type of bank accounts available to the public, as a simplifying assumption. We also assume that bank A is the only bank in the economy, or alternatively, that it describes the incumbent banking sector as a whole. We also assume a closed economy.

5 To be sure, money multiplication based on the funds raised at the start is also a possibility. While this would enlarge the balance sheet of the FinTech platform, the sequence of events would remain the same.

6 The US Lending Club is a platform that used to operate along these lines before turning into a licensed bank in 2020; Prestadero is another example of such platform from Mexico.

7 In the context of US mortgage lending, ‘FinTech’ encompasses all non-bank lending institutions whose credit making process is almost entirely done online (Buchak et al., 2018; Fuster et al. 2019).

## Appendix A

**Table A1.** Interview details.

Interview Code	Country	Sector	Position	Date
INT_1	Argentina	FinTech	Chief Executive Officer	03/04/2020
INT_2	Argentina	FinTech	Chief Executive Officer	18/03/2020
INT_3	Argentina	FinTech	Chief Executive Officer & Co-founder	25/03/2020
INT_4	Argentina	FinTech	Founder	27/03/2020
INT_5	Argentina	Consulting	Leading Partner	20/03/2020
INT_6	Argentina	FinTech	Chief Executive Officer	20/03/2020
INT_7	Argentina	Central Bank	Financial Innovation Manager (former)	26/03/2020
INT_8	Argentina	Central Bank	Board Member	27/03/2020
INT_9	Brazil	FinTech	Public Policy Director	20/05/2019
INT_10	Brazil	FinTech	Founder & Chief Executive Officer	24/05/2019
INT_11	Brazil	bank lobbying organization	Independent Board Member	20/05/2019
INT_12	Brazil	Central Bank	Executive (Banking Supervision Department)	23/05/2019
INT_13	Brazil	Brazilian Securities and Exchange Commission (CVM)	Superintendence of Market Relations	23/05/2019
INT_14	Brazil	International Bank	Treasury Director	22/05/2019
INT_15	Brazil	bank lobbying organization	Chief Economist	22/05/2019
INT_16	Mexico	FinTech	Chief Operating Officer	25/03/2020
INT_17	Mexico	bank lobbying organization	Coordinator of FinTech group	23/03/2020
INT_18	Mexico	Asset Management	Chief Economist	24/03/2020
INT_19	Mexico	Investment Banking	Head of Operations	25/03/2020
INT_20	Mexico	stock market	Executive (Investor Relations)	24/03/2020
INT_21	United States	Investment Banking, Accounting, Asset Management	Financial Advisor, Business Owners	12/03/2019
INT_22	United States	Asset Management	Vice Chairperson	11/04/2020
INT_23	United States	Management Consulting	Partner	10/04/2019
INT_24	United States	Consulting—Location Management	Executive	26/03/2019
INT_25	United States	Data and Financial Market Infrastructure	Director of Research	22/03/2019
INT_26	United States	FinTech	Co-founder and Chief Operating Officer	12/04/2019
INT_27	United States	Economic Development Agency	Executives	29/03/2019
